# Surgical treatment for heart failure: cell-based therapy with engineered tissue

**DOI:** 10.20517/2574-1209.2019.16

**Published:** 2019-09-17

**Authors:** Jordan J. Lancaster, Jen Watson Koevary, Ikeotunye Royal Chinyere, Sherry L. Daugherty, Kenneth A. Fox, Steven Goldman

**Affiliations:** 1 Sarver Heart Center, University of Arizona, Tucson, AZ 85724, United States; 2 Department of Medicine, University of Arizona, Tucson, AZ 85724, United States; 3 Department of Biomedical Engineering, University of Arizona, Tucson, AZ 85724, United States; 4 Department of Surgery, University of Arizona, Tucson, AZ 85724, United States

**Keywords:** Heart failure, induced pluripotent stem cells, tissue engineering

## Abstract

This review will outline cell-based therapy for heart failure focusing on tissue engineering to deliver cells to the damaged heart. We will present an overview of the central approaches focusing on pluripotent stem cell-derived cells, mechanisms of action, autologous *vs*. allogeneic cell approaches, immunologic modulation, and safety considerations. We will outline the progress that has been made to-date and define the areas that still need to be investigated in order to advance the field.

## INTRODUCTION

The ability to differentiate specialized functional cells from pluripotent stem cells (PSCs) has opened up the possibility of new therapeutic approaches that provide the functional units to solve the underlying causes of disease. Work using cells differentiated from embryonic PSCs first appeared 1998 when Jamie Thomson and colleagues published a report of deriving embryonic stem cell lines from human blastocyts^[1]^. This same laboratory described creating induced PSC lines from human somatic cells in 2007^[2]^. In 2012, the Nobel Prize in Physiology or Medicine was awarded to John B. Gurdon and Shinya Yamanaka for the discovery that mature adult differentiated somatic cells can be reprogrammed to a pluripotent state to create an unlimited source of any cell in the body. The ability to generate human induced PSC-derived cardiomyocytes (PSC-CMs) from adult cells has further broadened interest and study in the use of these cells in the heart. A number of investigators are now culturing these cells, developing methods to maintain differentiated cell phenotype, and studying healthy and diseased human cardiomyocytes^[3–6]^. Much of this work looks at reprogramming somatic cells from healthy and diseased donors to study known mutations and deficiencies of cardiomyocytes, as well as identify potential treatments. Hurdles regarding target identification and drug development have been attributed to variability in phenotype, thus the growing body of work in this area^[7,8]^.

Prior to the development of PSCs, the early work with cell-based therapy for acute myocardial infarction and chronic heart failure (CHF) involved administering varying adult somatic cell populations. With the exception of the skeletal myoblasts^[9]^, these approaches proved safe but had limited therapeutic efficacy with respect to improved cardiac function and structural remodeling^[10,11]^. With the advent of PSCs it became possible for investigators to create the functional building blocks of the heart allowing for the development of tissue engineering (TE) approaches, cellular reprogramming, and gene editing to optimize the delivery of tissue function specific cells to the damaged heart^[12–23]^.

## AUTOLOGOUS *VS*. ALLOGENEIC PREPARATIONS

Early in the evolution of cell-based therapies there was enthusiasm to use autologous approaches with the patient’s own cells so as to avoid immune rejection. However, it has become clear that limitations with autologous approaches exist: sample collection and preparation from individual patients is time-consuming, costly and may ultimately lack efficacy because of problems with cellular genome stability and regenerative potential. To overcome these limitations, allogeneic approaches have been adopted using screened, optimal donors. This donor vetting improves both the quality and potency of the cell product, increases cell availability, and decreases cost through scaled manufacturing. The paradigm switch from autologous to allogeneic was observed with the first clinical trial using PSC derived cells for age-related macular degeneration, which started with autologous preparations but subsequently changed to using allogeneic cells^[24]^.

## IMMUNE CONSIDERATIONS

Immunologic rejection is something relevant for all proposed cell-based therapies^[25]^. The concept of accounting for the immune system is based on the belief that PSC derived cells are not immune-privileged because of Human Leukocyte Antigen (HLA)/ABO antigens. Investigators have suggested that the expression of these antigens in PSC-derived cells equates them with any other organ transplants, all of which require immune suppression^[26]^. Immunosuppressive agents such as cyclosporine and tacrolimus have been used for solid organ transplant and described extensively in pre-clinical studies of TE preparations. However, they are costly, may be cytotoxic, and cause adverse effects. There is a clinical trial in Japan using PSC-CMs in patients with CHF patients where the patients receive non-HLA mapped allogeneic cardiomyocytes and are given immune suppression^[27]^. The use of HLA mapping is one option by which to circumvent immunosuppressive agents; allogeneic cell lines of HLA-typed donors could be generated to maximally match each recipient, though an identical match is statistically improbable. Such a strategy would require creating and maintaining large cell banks. Generating personalized products for individual patients from the bank would be expensive and require HLA typing of every patient. The ability to edit candidate therapeutic cell lines is also being explored, to make a hypo-immune cell, one that would be considered “universal”. Each cell line could be edited to remove HLAs while still maintaining markers of identity so as to go undetected as foreign by the immune system, theoretically eliminating the need for immunosuppression^[28]^. Gene editing may prove valuable in the future to generate cell therapy candidates that integrate or persist in the host without losing potency.

## ENGINEERED TISSUE TO TREAT HEART FAILURE

The development of cell-based TE approaches is exciting and in essence has the potential to generate a therapeutic tissue with properties and function of the myocardium^[13]^. Investigators have constructed tissue patches that could be manipulated with respect to anisotropic components, degree of electrical conductance, and mechanical qualities such as cardiomyocyte alignment and electromechanical coupling or using decellularized starting material as a substrate^[29,30]^. Cardiac patches have been engineered with nanotopographically-defined hydrogels meant to enhance cardiac regeneration by providing functional cell-material interfaces^[31]^. Three-dimensional (3-D) printing has also been utilized to generate TE scaffolds by organizing special patterning of stem cells. The 3-D generated patches proved sufficient to promote rapid vascularization with the potential to improve cardiac function and reverse maladaptive left ventricular (LV) remodeling^[32]^. Cardiac scaffolds have been made from electro-conductive acid-modified silk fibroinpoly (pyrrole) substrates. With PSC derived cardiomyocytes these grafts showed enhanced gap junction distribution and cardiomyocyte maturation^[33,34]^.

**Figure 1.**
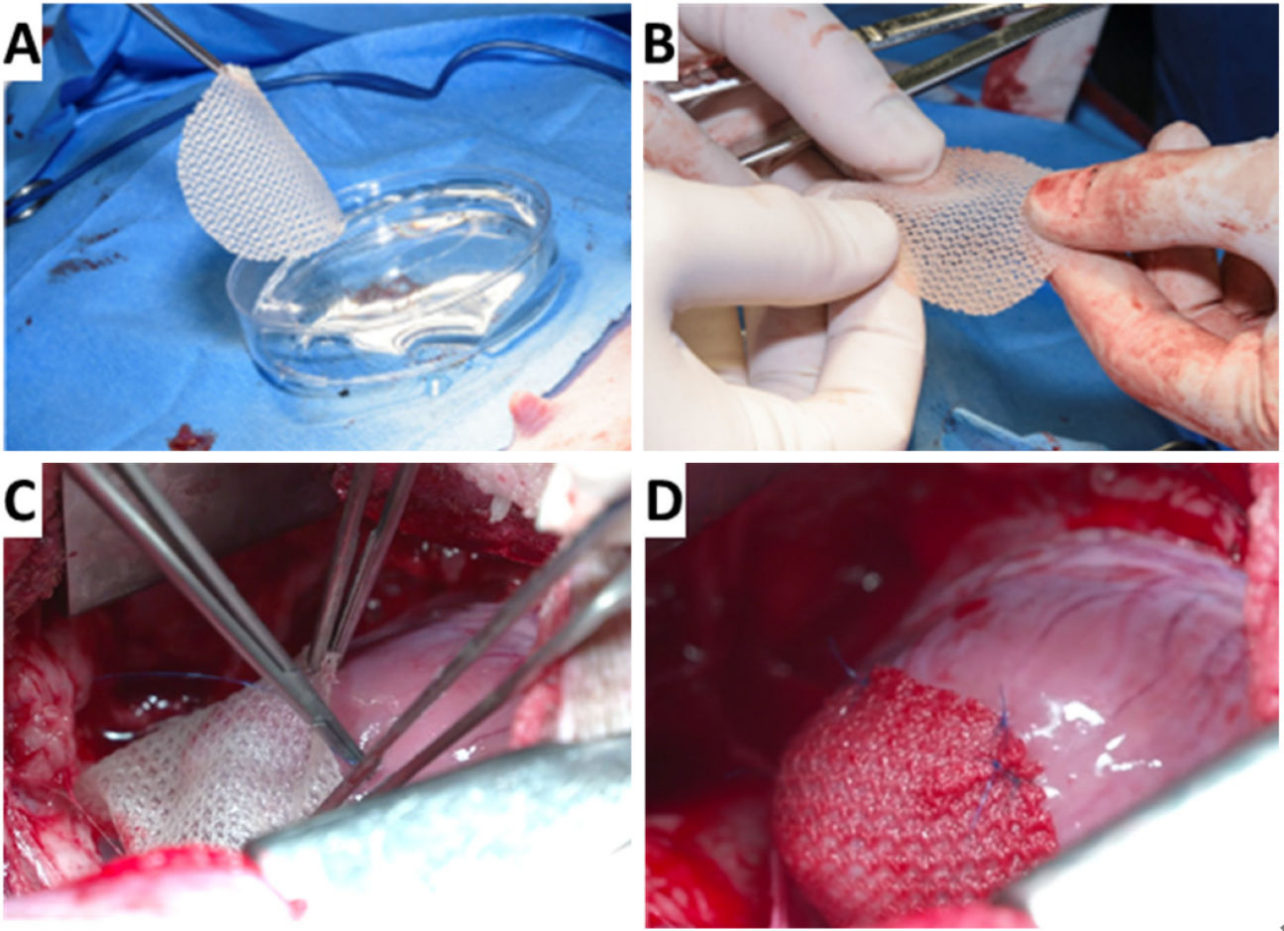
Intra-operative implant of cardiac patch in mini-swine one month after myocardial infarction. A: the patch is picked up by surgeon (KF) in the operating room; B: easily handled by surgeons prior to implantation; C: implanted in mini-swine through a mini median sternotomy; D: close up view of the patch successfully implanted on the heart

Lancaster *et al*.^[35]^ have developed a TE cardiac patch composed of a bioabsorbable scaffold embedded with human neonatal fibroblasts and PSC-CMs that is implanted on the epicardial surface of the heart to treat CHF. The fibroblasts proliferate and synthesize glycosaminoglycans, collagen, elastin, fibronectin and laminin while providing a secretome of growth factors and cytokines. The fibroblasts support the incorporation of the cardiomyocytes into the scaffold and the cardiomyocytes provide additional complementary secretomes. This cardiac patch increases myocardial blood flow, reverses maladaptive LV remodeling, activates endogenous growth factor secretion, recovers hibernating myocardium and improves LV function and improves electrical propagation through the previously scarred myocardium and enhances electrical stability of the healthy tissue-scar interfac^[14–16,35]^. The specific growth factors are angiopoietin-1, β-myosin heavy chain, connexin 43, insulin like growth factor and vascular endothelial growth. This patch is robust; it is easy to handle for open surgical or minimally invasive implantation. Figure 1 and the Supplementary Video 1 demonstrate implantation on the epicardium in an infarcted Yucatan mini swine via a mini median sternotomy where the patch is positioned to broadly cover all damaged tissue. We envision that multiple patches and/or multiple patch applications could be applied if needed. Table 1 summarizes previous work done with TE approaches using iPSC-CMs to treat heart failure. The epicardium may play an important role in regulating regeneration and may be the catalyst of the beneficial effects we and other investigators see when we implant cell-seeded scaffolds on the epicardium^[36–38,48–50]^.

Implanting TE scaffolds in preclinical animal models of heart failure has proven safe. The data have shown no teratoma formation, accompanied by hemodynamic benefits, and attenuated LV remodeling, however data show limited long-term engraftment with the implanted cells despite providing immune suppression^[20]^. Menasché *et al*.^[19]^ have transplanted PSC-derived cardiac progenitor cell fibrin patches in 6 patients with decreased LV function and showed that the treatment was safe. No arrhythmias were noted by ICD interrogations and no tumors were reported^[19]^. Based on this work, these investigators performed a second trial of 10 patients, but these results are still forthcoming. To date, there is only one clinical trial has commenced using PSC-CMs to treat patients in CHF; this trial is currently ongoing in Japan^[27]^. Cardiothoracic surgeons are implanting cell sheets on the epicardial surface of the heart in immune-suppressed patients. Table 1 summarizes some of the TE scaffolds developed to treat heart failure progressing from early work with rat neonatal cardiomyocytes to the clinical trial in Japan with PSC-CMs on cell sheets.

## MECHANISMS OF ACTION OF CELL-BASED THERAPY

It is likely that the mechanism of action that improves LV function with cell-based therapy is multi-modal and is shaped by the fate of the cells, as the cells can be intended as either integrating or non-integrating. Integrating cells directly replace lost myocardium and theoretically contribute to mechanical function. Non-integrating approaches are transient in that the cells persist for days, weeks or months, imparting a beneficial effect, before ultimately being cleared from the tissue. In both integrating and non-integrating approaches, a secretome of soluble factors such as growth factors, exosomes, and miRNAs are thought to be responsible for the beneficial effects^[48–50]^. The specific paracrine stimulation may vary by implanted cell type and may be modulated by the cell culture environment, delivery platform, and other relevant variables.

In pre-clinical studies Lancaster *et al*.^[14–16,35]^ did not immune suppress their animals and long-term physiologic benefits were seen when implanting human xenografts in immune-competent animals. The transplanted PSC-CMs did not persist beyond four weeks post-implantation, but the initial functional benefit continued to persist at ten weeks post-implantation^[35]^.

## SAFETY CONSIDERATIONS

The use of PSC-derived cells offer potential as a source for therapeutic advancements in all tissues, not just the heart. The safety of these cell preparations when implanted in patients is obviously important. The potential for tumorigenicity resulting from undifferentiated PSCs contaminants is a concern, particularly with integrating cell preparations. The Food and Drug Administration requires extensive preclinical testing for teratoma formation before approving PSC-derived preparations for clinical use. During somatic skeletal myoblasts injections into the myocardium, ectopic foci were established that generated VT from spontaneously depolarizing cells^[9]^. The finding of increased incidence of ventricular tachyarrhythmia has also been reported with human embryonic stem cell-derived cardiomyocyte injections into non-human primates and swine hearts^[51,52]^. Interestingly in preclinical studies with PSC-CMs TE scaffolds implanted on the epicardium, the animals remain in normal sinus rhythm without any arrhythmias^[20,35]^.

## PROSPECTS FOR THE FUTURE

Looking into the future to predict the next advances of TE approaches to treat CHF is difficult but while creating a cardiac patch has received a lot of interest, there are a number of other potential approaches. Investigators have proposed decellularizing entire hearts and then repopulating with new PSC-derived cells^[53]^. There are also efforts to use 3-D printing to print entire organs^[54]^. Synthesizing microvesicles such as exosomes or secretomes that are secreted from the PSC-derived cells are being explored as a way to bypass cell-based therapy and still retain the paracrine effect^[48]^. Some of the issues outlined previously need to be addressed such as the best strategies for immune suppression in patients, required maturity of PSC-derived cells, use of integrating or non-integrating approaches and duration of transplanted cell survival in order to result in the most beneficial effects. A recent review summarizes the “bottlenecks” for the future of TE cardiac scaffolds^[55]^. Despite these issues, we see a bright future for using TE approaches for regenerative cardiology and all of medicine. The FDA now has a rapid approval process for cell therapy and therapeutic TE products. The Regenerative Medicine Advanced Therapy designation of the 21st Century Cures Act is designed to help accelerate medical product development and bring new innovations and advancements to the patients who need them urgently^[56]^.

## CONCLUSIONS

The availability of PSC-derived cells and ability to generate TE approaches have introduced a unique opportunity to develop novel strategies to treat patients. While there remain points of concern that need to be addressed with respect to PSC-derived therapy, such as defining the mechanisms of action and the potential need for immune suppression the field as a whole is moving forward and TE surgical therapies for regenerative cardiology are closer to reality today than ever before.

## Supplementary Material

Supplementary Material

## Figures and Tables

**Table 1.** Summary of TE approaches using pluripotent derived cardiac cells for heart failure

Cell type	Auto/allo	Scaffold	Stage	Investigator^(Ref)^
Neonatal rat cardiomyocytes	Auto	EHT	Pre-clinical	Zimmerman *et al*.^[39]^
Neonatal rat cardiomyocytes	Auto	Bioabsorbable polymer	Preclinical	Lancaster *et al*.^[15,16]^
Vascular smooth muscle	Auto	PCLA	Pre-clinical	Matsubayashi *et al*.^[40]^
Skeletal myoblasts	Auto	Cell sheet	Clinical	Sawa *et al*.^[41]^
Skeletal myoblasts	Auto	Cell sheets	Clinical	Yoshikawa *et al*.^[42]^
ES derived cardiac progenitor	Allo	Fibrin	Clinical	Menasché *et al*.^[19]^
PSC-CMs	Allo	Cell sheets	Pre-clinical	Kawamura *et al*.^[20]^
PSC-CMs	Allo	EHT	Pre-clinical	Yorgan *et al*.^[43]^
Bone marrow stem cells	Auto	Fibrin	Pre-clinical	Liu *et al*.^[44]^
Mesenchymal progenitor cells	Allo	Fibrin	Pre-clinical	Godier-Furemont *et al*.^[45]^
PSC-CMs	Allo	3-D scaffold	Pre-clinical	Gao *et al*.^[17]^
ES cardiomyocytes/ progenitors	Auto	Fibrin	Pre-clinical	Liau *et al*.^[29]^
Human cardiac progenitor	Allo	hdECM	Pre-clinical	Jang *et al*.^[32]^
PSC-CMs	Allo	Silk fibrion-poly (pyrrole)	Pre-clinical	Tsui *et al*.^[33]^
PSC-CMs	Allo	Cell sheets	Pre-clinical	Matsuura *et al*.^[46]^
PSC-CMs	Allo	Cell Sheets	Pre-clinical	Sasagawa *et al*.^[47]^
PSC-CMs	Allo	Bioabsorbable polymer	Pre-clinical	Lancaster *et al*.^[35]^
PSC-CMs	Allo	Cell sheets	Clinical	Cyranoski/Sawa^[27]^

TE: tissue engineering; Allo: allogeneic; Auto: autologus; hdECM: decellularized extracellular matrix; ES: embryonic stem; EHT: engineered heart tissue; PSC-CMs: human induced pluripotent stem cell-derived cardiomyocytes; PCLA:sponge polymer of epsilon-caprolactone-co-L-lactide reinforced with knitted poly-L-lactide fabric
